# Autoantibodies to Osteoprotegerin are Associated with Low Hip Bone Mineral Density and History of Fractures in Axial Spondyloarthritis: A Cross-Sectional Observational Study

**DOI:** 10.1007/s00223-017-0291-2

**Published:** 2017-05-22

**Authors:** Barbara Hauser, Sizheng Zhao, Micaela R. Visconti, Philip L. Riches, William D. Fraser, Isabelle Piec, Nicola J. Goodson, Stuart H. Ralston

**Affiliations:** 1 Centre for Genomics and Experimental Medicine, Institute of Genetics and Molecular Medicine, University of Edinburgh, Edinburgh, UK; 2 Department of Academic Rheumatology, Aintree University Hospital, Liverpool, L9 7AL UK; 3 Musculoskeletal Biology I, Institute of Ageing and Chronic Disease, University of Liverpool, Liverpool, UK; 4 Department of Medicine, University of East Anglia, Norwich, UK

**Keywords:** Ankylosing spondylitis, Axial spondyloarthritis, Osteoporosis, Bone mineral density, Osteoprotegerin

## Abstract

Osteoporosis is a recognised complication of axial spondyloarthritis (axSpA) and is thought to be due to functional impairment and the osteoclast-activating effects of proinflammatory cytokines. The development of autoantibodies to OPG (OPG-Ab) has been associated with severe osteoporosis and increased bone resorption in rheumatoid arthritis. In this study, we screened for the presence of OPG-Ab in axSpA and reviewed their clinical significance. We studied 134 patients, recruited from two centres in the United Kingdom. Their mean age was 47.5 years and 75% were male. Concentrations of OPG-Ab were related to bone mineral density (BMD) and fracture history using linear and logistic regression models adjusting for age, gender, disease duration and activity, body mass index and bisphosphonate use. We detected OPG-Ab in 11/134 patients (8.2%). Femoral neck and total hip BMD were significantly reduced in OPG-Ab positive patients (0.827 vs. 0.967 g/cm^2^, *p* = 0.008 and 0.868 vs. 1.028 g/cm^2^, *p* = 0.002, respectively). Regression analysis showed that the presence of OPG-Ab was independently associated with total hip osteopenia (OR_adj_ 24.2; 95% CI 2.57, 228) and history of fractures (OR_adj_ 10.5; 95% CI 2.07, 53.3). OPG-Ab concentration was associated with total hip BMD in g/cm^2^ (*ß* = −1.15; 95% CI −0.25, −0.04). There were no associations between OPG-Ab concentration and bone turnover markers, but free sRANKL concentrations were lower in OPG-Ab-positive patients (median 0.04 vs. 0.11 pmol/L, *p* = 0.050). We conclude that OPG-Ab are associated with hip BMD and fractures in axSpA suggesting that they may contribute to the pathogenesis of bone loss in some patients with this condition.

## Introduction

Axial spondyloarthropathy (axSpA) is characterised by inflammation of the sacroiliac joints and entheses. Disease progression involves not only new bone formation at sites of inflammation but also, paradoxically, an increased risk of osteoporosis and vertebral fractures [1–3]. Osteoporosis is present in up to 25% of patients [4] and vertebral fractures have been reported in up to 30% of patients [5, 6]. Both can occur in early disease [7, 8]. In contrast to the general population, axSpA-associated osteoporosis is more prevalent in men and occurs at younger ages [8], which limits the value of conventional fracture risk assessment tools such as FRAX^®^ [9].

AxSpA shares many factors in common with other inflammatory arthropathies that are associated with accelerated bone loss, including impaired physical mobility [8] and increased production of cytokines such as TNF and IL17 which stimulate osteoclastic bone resorption [10]. Although the patterns of bone involvement differ in rheumatoid arthritis (RA) and axSpA, the final common pathway of bone loss in both cases is increased osteoclastic bone resorption which is critically dependent on the stimulatory effects of RANKL, counterbalanced by the inhibitory effects of OPG [11]. However, axSpA also differs in that it has younger age of onset, male predominance and lower systemic inflammatory burden. Other novel factors may therefore be associated with the development of premature osteoporosis and fractures in the axSpA population.

Autoantibodies to osteoprotegerin (OPG-Ab) were previously identified as a cause of high turnover osteoporosis in a patient with coeliac and autoimmune thyroid disease [12]. OPG-Abs have also been detected in patients with RA, where they were associated with increased bone resorption [13] and in coeliac disease where they were associated with reduced BMD [14]. The aim of this study was to determine if OPG-Abs were present in axSpA and to explore whether they were associated with BMD or history of fractures.

## Methods

We enrolled consecutive axSpA patients who presented to specialist rheumatology clinics at Aintree University Hospital in Liverpool and the Western General Hospital in Edinburgh, between July 2011 and February 2015. Patients were included in the study if they met the modified New York criteria for ankylosing spondylitis [15] and/or the ASAS criteria for axSpA [16].

We recorded demographic information, disease characteristics (age at diagnosis, HLA-B27 status if available, extra-axial involvement), and current medication [NSAIDs, DMARDs, calcium/vitamin D, bisphosphonates and TNF inhibitors (TNFi)], ever-smoking, current glucocorticoid use and body mass index (BMI). We enquired about a history of clinical fractures and fracture site (vertebral or non-vertebral). Clinical notes and radiographs were reviewed to verify self-reported fractures. Disease duration was defined as the number of years since first diagnosis. Disease activity was assessed using the Bath Ankylosing Spondylitis Disease Activity Index (BASDAI), spinal pain visual analogue scale (spVAS) and Bath AS Functional Index (BASFI). Blood samples were obtained for erythrocyte sedimentation rate (ESR) and C-reactive protein (CRP) using the local hospital laboratories. Serum samples were also prepared at the time of enrolment and stored at −80 °C until use for measurement of specialised markers of bone turnover and OPG-Ab as described below.

Bone mineral density (BMD) was assessed using anteroposterior DXA of the lumbar spine (L1–L4), femoral neck and total hip using Lunar iDXA (GE Healthcare) at Liverpool and Hologic QDR4500 at Edinburgh. Hologic BMD values in g/cm^2^ were converted to Lunar equivalents as manufacturer's recommendations [17, 18]. Osteopenia and osteoporosis were defined as *T*-scores <−1 and ≤−2.5, respectively [19].

Measurement of OPG-Ab was performed at the Centre for Genomic and Experimental Medicine by an indirect ELISA developed in-house as described previously [14]. A standard curve comprising serial dilutions of serum from the index case was run on each plate with an arbitrary value of 100 units assigned to neat serum. The concentrations of OPG-Ab were estimated from this standard curve. Each sample was run in triplicate and repeated at least once on a different day. One result of each triplicate was discarded as an outlier if it was more than three standard deviations outside the mean of two most concordant values, except where the two most concordant values were identical. The mean of the remaining values was used for analysis. Inter- and intra-assay coefficients of variation (CV) for positive OPG-Ab ELISA titre were 16.6 and 17.3%, respectively. A previous study of 100 healthy controls [mean age 57.8 (SD ± 12)] with normal BMD demonstrated a mean titre of 3.46 (SD ± 3.64) and was used to define the limit for OPG-Ab positivity [14]. Positive OPG-Ab was defined as titres three standard deviations above the mean [20], which equates to ≥14.3 units.

Measurements of OPG, free soluble RANKL (sRANKL), the N-terminal pro-peptide of type I collagen (PINP) and the C-terminal type 1 crosslinked telopeptide (CTX) were performed at the University of East Anglia bioanalytical facility. Measurements of CTX (ng/ml) and PINP (ng/ml) were performed on COBAS 6000 (Roche Diagnostics) with an inter-assay CV of 3.9 and 4.0%, respectively. Concentrations of OPG (pmol/L) and free sRANKL (pmol/L) were measured using ELISA kits (Biomedica, Austria) following manufacturer’s instructions. Performance of these kits (on duplicated samples) was intra-assay CV 3.1% for OPG and 3.8% for sRANKL. Inter-assay CV were 6.6% for OPG (*n* = 4) and 4.1% for sRANKL (*n* = 3) across the standard curve concentrations for the assay. Reference ranges for sRANKL, CTX and PINP were obtained from the manufacturer literature, and for OPG from the population study [21]: OPG (male 0.124–10.690 pmol/L; female 0–11.475 pmol/L), sRANKL (male 0.022–0.381 pmol/L; female 0.020–0.329 pmol/L), CTX (male 0.158–0.442 ng/ml; female 0.092–0.436 ng/ml) and PINP (male 20–76 ng/ml; female 15–59 ng/ml).

Statistical analyses were performed using Stata13. Data from both study sites were combined for analyses. Comparative analysis was performed using Mann–Whitney *U* test for non-normally distributed variables, Student’s *t* test for normally distributed variables and Fisher’s exact test for categorical variables. Associations between OPG-Ab concentration and OPG, free sRANKL, CTX and PINP were assessed using Spearman’s rank correlation in the total cohort and separately in patients who were not receiving bisphosphonates. Association between positive OPG-Ab and disease characteristics was assessed with univariate logistic regression. Correlation between BMD (g/cm^2^) and OPG-Ab concentration was demonstrated using scatter graphs.

In the first instance, regression models were used to assess associations between measures of bone density and OPG-Ab *positivity* as the independent variable. Linear models were used for BMD (g/cm^2^), *T*- and Z-scores as dependent variables, adjusted for age (not included in *Z*-score model), gender, disease duration, bisphosphonate use, BASDAI and BMI. Logistic models were used for osteopenia, osteoporosis and fractures as dependent variables, using the same covariates. No imputation was performed for missing data; therefore, multivariable models were limited to a subgroup with complete data for all covariates. In addition to analyses of OPG-Ab *positivity*, the same linear and logistic models were generated using OPG-Ab *concentration* as the independent variable. Residuals of linear models were tested against a normal distribution using kernel density plots and skewness–kurtosis test. Finally, backward stepwise regression analyses were performed to identify independent explanatory variables for total hip, femoral neck and spine g/cm^2^ each as the dependent variable, using *p* > 0.1 as criteria for removal from the full model. Significant association was defined as *p* < 0.05.

## Results

The axSpA cohort comprised 134 patients: 53 from Edinburgh and 81 from Liverpool. There were no significant differences in patient or disease characteristics between the two study sites (data not shown). The median OPG-Ab titre was 2.8 (range 0.2, 36; interquartile range [IQR] 1.4, 4.3). A positive OPG-Ab result was detected in 11 patients (8.2%). There was no difference in proportion of positive OPG-Ab between study sites (*p* = 0.526). Demographics and disease characteristics of patients positive and negative for OPG-Ab are shown in Table 1. The cohort was predominantly male (75%) with a mean age of 47.5 years (SD ± 15) and medium disease duration of 6.4 years [IQR 1.9, 17.8]. Patients positive for OPG-Ab were older and had longer disease duration. They were also lower in height, although BMI was similar between the groups.

**Table 1:** Patient and disease characteristics by OPG-Ab status

	Positive *n* = 11	Negative *n* = 123	*p*-value
Demographics			
Age (years)	56.1 ± 15.5	46.2 ± 14.7	0.036
Males	8 (73%)	92 (75%)	1.000
HLA-B27 (*n* = 59)	3 (60%)	39 (72%)	0.620
Age diagnosed	43.3 ± 15.1	40.9 ± 14.5	0.610
Median years disease duration	22.3 [0.8, 33.1]	5.6 [1.9, 16.3]	0.067
Ever smokers	5 (45%)	40 (33%)	0.510
Height (cm) (*n* = 130)	164 ± 7.5	171 ± 9.6	0.018
Weight (kg) (*n* = 132)	78.6 ± 14.5	81.4 ± 17.3	0.609
BMI (kg/m^2^) (*n* = 130)	29.3 ± 5.9	27.8 ± 5.8	0.391
Disease activity			
Median BASDAI (*n* = 132)	3.9 [2, 5.7]	5.5 [3.2, 7.7]	0.137
Median spVAS (*n* = 126)	5 [2, 8]	6 [2.9, 8]	0.741
Median BASFI (*n* = 106)	4 [3.4, 8.5]	5.3 [3.4, 7.4]	0.869
Median ESR (mm/h) (*n* = 111)	14 [5, 27]	8 [5, 17]	0.420
Median CRP (mg/L) (*n* = 109)	1 [1, 6]	4 [1, 9]	0.230
Extra-axial involvement			
Peripheral joint involvement	4 (36%)	42 (34%)	1.000
Psoriasis	2 (18%)	18 (15%)	0.671
Uveitis	2 (18%)	44 (36%)	0.329
Inflammatory bowel disease	1 (9%)	14 (11%)	1.000
Medication			
TNF inhibitor	7 (64%)	38 (31%)	0.043
Synthetic DMARD	0	15 (12%)	0.256
NSAIDs	6 (55%)	77 (65%)	0.520
Glucocorticoid	1 (9%)	2 (2%)	0.228
Bisphosphonates	4 (36%)	7 (6%)	0.006
Calcium and vitamin D	7 (64%)	57 (46%)	0.271
Lumbar spine^a^			
g/cm^2^	1.226 ± 0.189	1.231 ± 0.235	0.947
*T*-score	0.2 ± 1.5	0.1 ± 2.0	0.963
Femoral neck^b^			
g/cm^2^	0.827 ± 0.131	0.967 ± 0.166	0.008
*T*-score	−1.68 ± 1.04	−0.57 ± 1.22	0.004
Osteopenia	9 (82%)	37 (35%)	0.007
Osteoporosis	3 (27%)	6 (6%)	0.039
Total hip^b^			
g/cm^2^	0.868 ± 0.142	1.028 ± 0.157	0.002
*T*-score	−1.5 ± 1.0	−0.3 ± 1.1	0.001
Osteopenia	9 (82%)	30 (28%)	0.001
Osteoporosis	2 (18%)	1 (1%)	0.023
History of fractures	7 (64%)	28 (23%)	0.007

Data shown as mean ± standard deviation; median [interquartile range]; number (percentage)

*BASDAI* Bath Ankylosing Spondylitis Disease Activity Index, *spVAS* spinal pain visual analogue scale, *BASFI* Bath AS Functional Index, *BMD* bone mineral density, *OPG-Ab* anti-OPG antibody, *n* number of available data)

^a^Measures of spine BMD were missing for one patient due to significant aortic calcification

^b^Measures of hip BMD were missing for two patients due to bilateral hip replacements

The BASDAI scores were generally high (median 5.4) with no significant difference between the OPG-Ab positive and negative groups. Peripheral joint involvement was reported in 35% of patients and 11% had associated inflammatory bowel disease. A third of patients were treated with TNFi and 64% were on NSAIDs at the time of recruitment. A greater proportion of patients positive for OPG-Ab were currently using TNFi (*p* = 0.043) and bisphosphonates (*p* = 0.006).

Bone density measurements were available in 120 patients, measured within a median of 0.2 months [IQR −9.6, 2.9] from the study visit. Existing DXA results were available for 50% of patients and a further 50% had scans organised at the time of the study assessment. Most scans (79%) were performed within 12 months of the study assessment. Spinal BMD was not measured for one patient due to significant aortic calcification. Hip BMD was not measured in two patients who had bilateral hip replacements. Differences between patients positive and negative for OPG-Ab are shown in Table 1. No significant differences in BMD were observed at the spine. Total hip and femoral neck BMD (g/cm^2^) and T-scores were all significantly lower in OPG-Ab positive patients. The same was true for Z-score (data not shown). Osteopenia of the total hip and femoral neck was significantly more common in OPG-Ab-positive patients (82 vs. 28%, *p* < 0.001 and 82 vs. 35%, *p* = 0.007, respectively). The same was true for osteoporosis of total hip and femoral neck (18 vs. 1%, *p* = 0.023 and 27 vs. 6%, *p* = 0.039, respectively).

A history of fracture was reported by 35 (26%) patients. Three patients (2%) had multiple vertebral fractures and 32 (24%) had non-vertebral fractures. OPG-Ab positive patients had a higher proportion of self-reported fractures (*p* = 0.007). Radiographs and/or clinical notes were available to confirm 17 of the self-reported fractures.

**Fig. 1:**
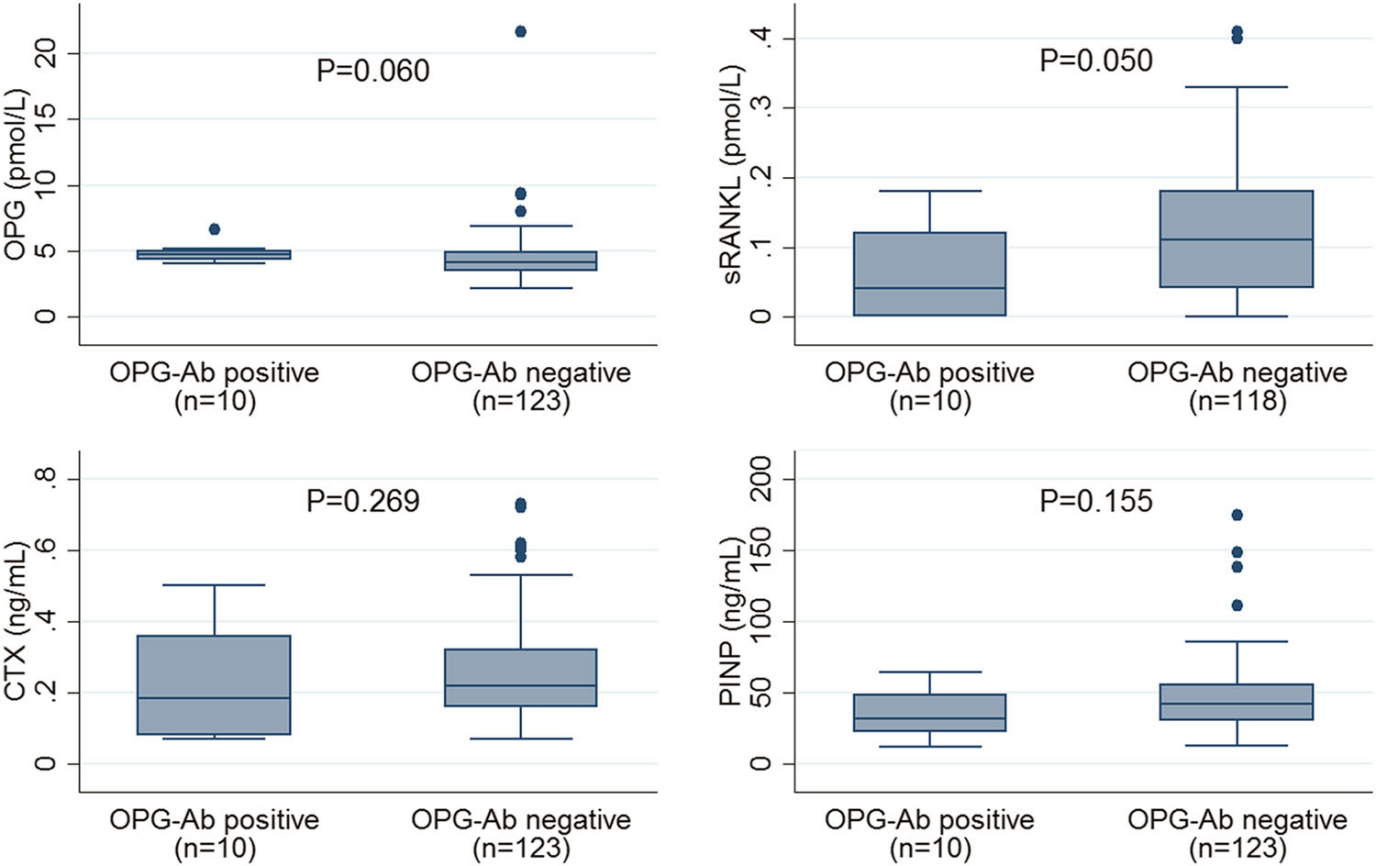
Biochemical markers of bone metabolism and OPG antibody status

**Fig. 2:**
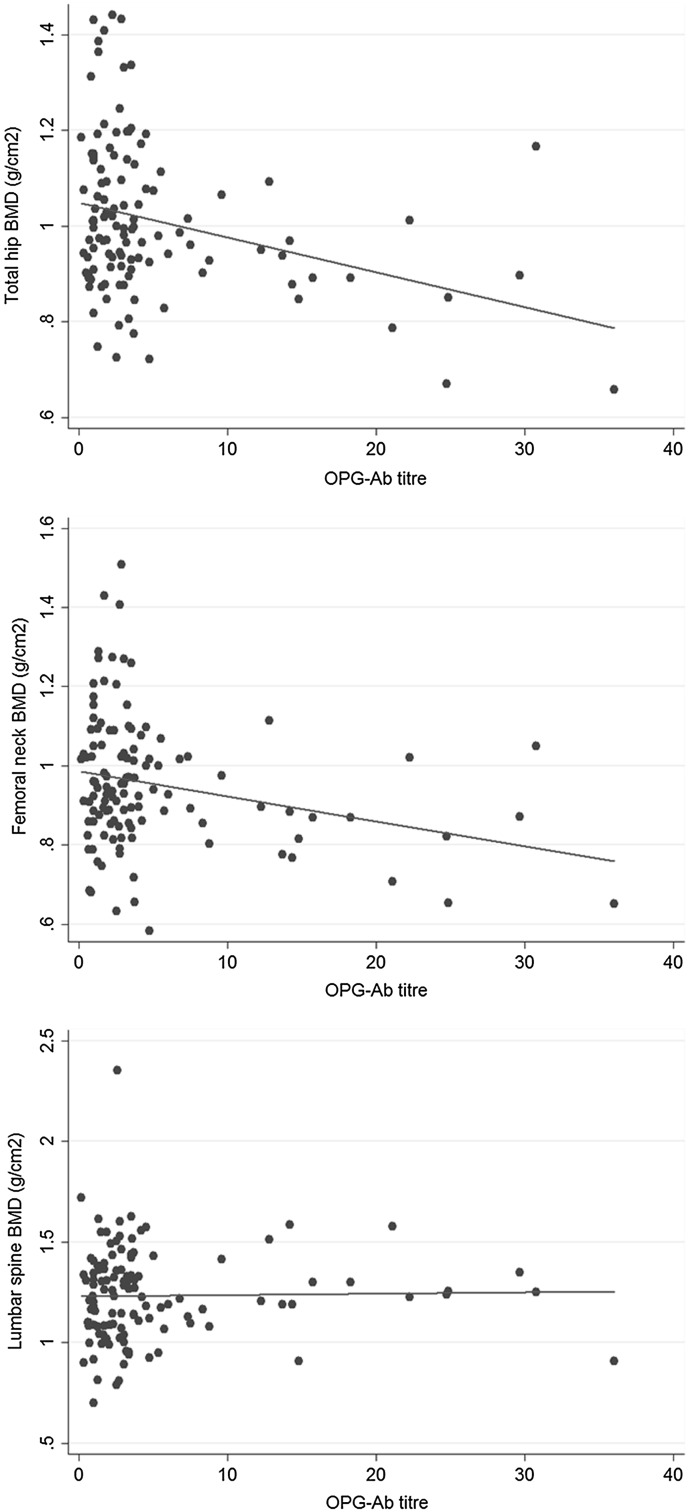
Scatter plot of anti-OPG antibody concentration against bone mineral density (g/cm^2^) of total hip, femoral neck and lumbar spine. *Lines* show least square *lines* of best fit

In the whole study cohort, the median level of OPG was 4.2 pmol/L [IQR 3.4, 4.9], sRANKL 0.11 pmol/L [IQR 0.04, 0.18], CTX 0.22 ng/mL [IQR 0.16, 0.32] and PINP 42 ng/mL [IQR 30, 55]. For OPG, 99% of the cohort were within the reference range, 77% for sRANKL, 71% for CTX and 88% for PINP. Serum concentrations of OPG, sRANKL, PINP and CTX in relation to OPG-Ab status are shown in Fig. 1. Concentrations of free sRANKL were lower in OPG-Ab positive group (*p* = 0.050) but there was no significant difference between the groups for concentrations of OPG, NTX or PINP. An inverse correlation was observed between OPG-Ab concentration and total hip and femoral neck BMD but not for spine BMD (Fig. 2). Similar correlations were observed for *Z*-scores (not shown).

Univariate logistic regression demonstrated significant associations between OPG-Ab positivity and disease duration (OR 1.05; 95% CI 1.01, 1.10) and age (OR 1.04; 95% CI 1.00, 1.09). Multi-adjusted regression models showed significant associations between positive OPG-Ab status and total hip BMD (g/cm^2^), *T*- and *Z*-scores (Table 2). Similar trends were observed for femoral neck BMD but significance was reached only for the association between femoral neck Z-score and OPG-Ab positivity. Positive OPG-Ab status was strongly associated with osteopenia at the total hip (OR_adj_ 24.2; 95% CI 2.57, 228) and femoral neck (OR_adj_ 21.3; 95% CI 1.96, 231) but not osteoporosis. In contrast, there was no association between OPG-Ab and lumbar spine BMD (g/cm^2^), *T*- or *Z*-scores. OPG-Ab positivity was significantly associated with history of self-reported fractures (OR_adj_ 10.5; 95% CI 2.07, 53.3). In regression models of OPG-Ab concentration, the same associations were demonstrated for measures of total hip BMD, osteopenia and previous fractures (Table 2). Post hoc analyses including TNFi as additional covariates in these models did not alter the effect sizes, and model comparisons using likelihood ratio test were not significant (data not shown). Residuals from linear models were normally distributed. Stepwise regression revealed that OPG-Ab concentration, age, gender and BMI were independently and significantly associated with total hip BMD (g/cm^2^). In contrast, only age and bisphosphonate use were associated with femoral neck BMD (g/cm^2^), while age, gender and BMI were associated with spinal BMD (g/cm^2^).

**Table 2:** Multivariable linear and logistic regression models demonstrating associations between BMD and fractures and OPG-Ab, adjusted for age, gender, disease duration, bisphosphonate use, BASDAI and BMI (age was not included for Z-score models)

	OPG-Ab positivity			OPG-Ab concentration		
	Effect size	95% confidence interval	*R* ^2^/pseudo *R* ^2^	Effect size	95% confidence interval	*R* ^2^/pseudo *R* ^2^
Total hip BMD (g/cm^2^)	***β*** **=** **−0.15**	**−0.25**, **−0.04**	0.26	***β*** **=** **−0.01**	**−0.01**, **−0.00**	0.26
Total hip *T*-score	***β*** **=** **−1.06**	**−1.82**, **-0.30**	0.21	***β*** **=** **−0.05**	**−0.09**, **−0.02**	0.22
Total hip *Z*-score	***β*** **=** **−1.12**	**−1.88**, **−0.36**	0.11	***β*** **=** **−0.05**	**−0.09**, **−0.02**	0.11
Femoral neck BMD (g/cm^2^)	*β* = −0.09	−0.20, 0.02	0.25	*β* = −0.00	−0.01, 0.00	0.25
Femoral neck *T*-score	*β* = −0.72	−1.52, 0.07	0.27	*β* = −0.03	−0.07, 0.00	0.27
Femoral neck Z-score	***β*** **=** **−0.79**	**−1.58**, **−0.01**	0.11	*β* = −0.03	−0.07, 0.00	0.10
Lumbar spine BMD (g/cm^2^)	*β* = −0.06	−0.21, 0.08	0.27	*β* = −0.00	−0.01, 0.00	0.27
Lumbar spine *T*-score	*β* = −0.47	−1.69, 0.76	0.23	*β* = −0.02	−0.07, 0.04	0.23
Lumbar spine *Z*-score	*β* = −0.73	−2.00, 0.55	0.21	*β* = −0.02	−0.08, 0.04	0.21
Total hip osteopenia (*T*-score <−1)	**OR**_**adj**_ **24.2**	**2.57**, **228**	0.19	**OR**_**adj**_ **1.19**	**1.07**, **1.31**	0.21
Total hip osteoporosis (*T*-score ≤−2.5)	OR_adj_ 45.3	0.22, 9521	0.61	OR_adj_ 1.11	0.92, 1.34	0.57
Femoral neck osteopenia (*T*-score <−1)	**OR**_**adj**_ **21.3**	**1.96**, **231**	0.25	**OR**_**adj**_ **1.17**	**1.05**, **1.30**	0.26
Femoral neck osteoporosis (*T*-score ≤−2.5)	OR_adj_ 1.41	0.08, 26.4	0.55	OR_adj_ 1.01	0.89, 1.13	0.55
History of previous fracture	**OR**_**adj**_ **10.5**	**2.07**, **53.3**	0.10	**OR**_**adj**_ **1.09**	**1.01**, **1.17**	0.08

Statistically significant models are in bold. Complete data for all covariates were available for 116 in the femoral neck and total hip models. Complete data for all covariates of the fracture model were available for 128

*BMD* bone mineral density, *OPG-Ab* anti-OPG antibody

No associations were observed between positive OPG-Ab status or concentration and markers of disease activity (data not shown). OPG-Ab concentration was associated with OPG (*ρ* = 0.17, *p* = 0.046) but not with sRANKL. There were no associations between OPG-Ab and CTX/PINP in the total cohort, nor in the subgroup not receiving bisphosphonates. sRANKL was associated with OPG (*ρ* = −0.38, *p* < 0.001) and PINP (*ρ* = 0.19, *p* = 0.032), and PINP was associated with CTX (*ρ* = 0.70, *p* < 0.001). Serum concentrations of OPG, sRANKL, CTX and PINP were not associated with fractures, hip or spine BMD (data not shown).

## Discussion

This cross-sectional study has demonstrated that 8.2% of patients with established axSpA were positive for OPG-Ab. These autoantibodies were independently associated with reduced hip BMD and a history of self-reported fractures. Adjusted for confounders, regression models showed that patients positive for OPG-Ab had lower total hip BMD by 0.15 g/cm^2^ or 1 unit in *T*-score than those who were negative. For each unit increase in OPG-Ab concentration, total hip *T*-score was 0.05 unit lower. Participants positive for OPG-Ab had 24-fold increased likelihood of hip osteopenia and tenfold increased likelihood of reporting a prior fracture.

The prevalence of positive OPG-Ab is similar to that reported in RA (9.3%) and coeliac disease (9.8%) but is significantly higher than that reported in healthy controls (1–1.4%) [13, 14]. This was unexpected since axSpA is not classically associated with the development of autoantibodies.

Previous studies have demonstrated associations between OPG-Ab and severe osteoporosis and fractures in a patient with coeliac disease and hypothyroidism, as well as increased bone resorption in RA and low BMD in coeliac disease [12–14]. However, this is the first study to show that positive OPG-Ab is associated with reduced hip BMD in axSpA. The lack of association with spine BMD may reflect syndesmophytes increasing anteroposterior BMD, even though there may be osteoporosis affecting the trabecular compartment of the vertebrae [1]. Future studies should utilise lateral DXA scans or quantitative CT to investigate the vertebral trabecular compartment BMD in relation to OPG-Ab in axSpA.

Since previous studies have shown that OPG-Abs block the inhibitory effect of OPG on RANKL-induced NFκB signalling in vitro, this provides an explanation for the association between OPG-Ab and bone loss [12, 13]. The findings reported here suggest that these antibodies may also contribute to the pathogenesis of systemic bone loss in some axSpA patients. Nonetheless, we acknowledge that OPG-Abs are likely to be only one of many contributory factors to the pathogenesis of bone loss in this complex disease.

In order to explore the pathophysiological mechanisms by which OPG-Ab were associated with reduced BMD in this population, we studied the relation between biochemical markers of bone resorption and bone formation and OPG-Ab, as well as possible associations between OPG-Ab and levels of OPG and free sRANKL. We were surprised to find a positive association between circulating levels of OPG and OPG-Ab. This has not been observed before [22] but a possible explanation might be that in some axSpA cases, local or systemic release of OPG may trigger an OPG-Ab response. We also found a negative correlation between OPG-Ab and free sRANKL concentrations. This is likely to be due to interference of OPG-Ab with the Biomedica sRANKL assay since this ELISA uses OPG as the capture antigen. It is therefore likely that OPG-Ab binds to the capture antigen, and in doing so blocks its ability to bind to free sRANKL. We found no correlation between OPG-Ab and CTX or PINP concentrations although this may have been due to the fact that one-third of patients with OPG-Ab were on bisphosphonates for the treatment of their osteoporosis. Further studies in untreated patients with axSpA would be warranted to determine if OPG-Ab are associated with increased bone turnover as has been reported previously [12, 13].

Patients positive for OPG-Ab were older and had longer disease duration. It is known that autoantibody production increases with age. However, associations in this study remained significant after adjusting for both age and disease duration. An association was found between OPG-Ab positivity and increasing disease duration, which was also observed in RA [13]. The reason for this association remains unexplained but we hypothesise that a cycle of chronic inflammation, B and T-cell proliferation and chronically increased RANKL/OPG concentrations may increase the risk of breaking tolerance and promote autoantibody production. Longitudinal studies are required to explore the timing or triggering factor of OPG-Ab development during the course of disease. Height was lower in patients positive for OPG-Ab compared with those who were negative. This may have been explained by their older age, but another possibility would be the increased prevalence of vertebral fractures in this group. No associations were demonstrated between OPG-Ab and disease activity or inflammatory markers which suggest that OPG-Ab may be independent of local inflammation in axSpA.

This small cohort had variable exposures to non-biological DMARDs and NSAIDs. While no association with these drug exposures was observed, the study was not adequately powered to explore such associations with OPG-Ab development. The association between TNFi use and OPG-Ab requires further exploration. Patients commenced on TNFi would have higher disease activity and inflammatory burden, which may promote autoantibody formation. Low BMD and fracture history were more prevalent in OPG-Ab-positive patients, which would explain increased bisphosphonate use in this group. A similar trend for increased TNFi and bisphosphonate use was seen in OPG-Ab-positive RA patients [13].

There were several limitations in this study. The association with fractures, although statistically significant, was based on a small number of events which are reflected by the wide confidence intervals. The intervals between measurement of OPG-Ab and BMD were variable. Although the median time between recruitment and DXA scan was 0.2 months, we included BMD results from up to four years before and one year after recruitment. We do not think that this would have substantially altered results since BMD is likely to have been relatively stable in this cohort of patients with well-established axSpA. In this regard, it has previously been found that accelerated bone loss predominantly occurs in early disease [7] and so it is likely that, in this population where median disease duration was 6.4 years, BMD results would have been stable. Another limitation is that the findings were primarily based on male axSpA patients who are more commonly affected by accelerated bone loss and vertebral fractures [8]. Due to the small number of female patients, it was not possible to evaluate the impact of gender on OPG-Ab positivity. Given most patients had established disease, it is important to emphasise that the results cannot be extrapolated to newly diagnosed younger patients who are at relatively higher risk of osteoporotic fractures [7]. A potential limitation was that not all self-reported fractures could be validated but this is unlikely to have resulted in systematic bias since fracture assessments were completed before the measurements for OPG-Ab were performed. It is of interest that the proportion of vertebral fractures in this cohort was lower than that reported in some historical cohorts [2]. This may reflect improved management or may have been due to the fact that lateral spine imaging was only performed in patients with symptoms suggestive of a clinical vertebral fracture. Because of that it is entirely possible that morphometric fractures may have not been detected.

In conclusion, the results of this study raise the possibility that OPG-Ab may play a role in axSpA-associated systemic bone loss in some patients and that these antibodies may be a biomarker for risk of bone loss and fractures. However, a causative role cannot be confirmed by this cross-sectional study. Larger longitudinal studies are needed to establish and quantify the impact of OPG-Ab, their functionality, and to explore the potential of early detection and prevention of OPG-Ab associated osteoporosis.
